# Detection and Molecular Characterization of *Giardia duodenalis* in Children Attending Day Care Centers in Majadahonda, Madrid, Central Spain

**DOI:** 10.1097/MD.0000000000000075

**Published:** 2014-09-26

**Authors:** Marta Mateo, María Mateo, Ana Montoya, Begoña Bailo, José M. Saugar, María Aguilera, Isabel Fuentes, David Carmena

**Affiliations:** Veterinary Faculty (Marta Mateo, AM), Alfonso X El Sabio University; Quirón Madrid University Hospital (María Mateo), European University of Madrid; and Parasitology Service (BB, JMS, MA, IF, DC), National Centre for Microbiology, Health Institute Carlos III, Majadahonda, Madrid, Spain.

## Abstract

Infections by the protozoan enteroparasites *Giardia duodenalis* and *Cryptosporidium* spp are a major cause of morbidity in children attending day care facilities in developed countries. In this cross-sectional study, we aimed to estimate the occurrence and genotype frequencies of these pathogens in children attending day care centers in Majadahonda, Central Spain. To do so, single stool samples were obtained from 90 children and tested for the presence of *G duodenalis* and *Cryptosporidium* spp by conventional microscopy and immunochromatography. Positive results by these techniques were subsequently confirmed by immunofluorescence microscopy. *G duodenalis*-positive samples were subjected to molecular characterization studies by multilocus sequence-based genotyping of the glutamate dehydrogenase and β-giardin genes of the parasite. *G duodenalis* assemblages were confirmed by restriction fragment length polymorphism analyses and sequencing. A socioepidemiological questionnaire was used to identify variables potentially associated with giardiasis/cryptosporidiosis in the population of children under investigation. Overall, *G duodenalis* and *Cryptosporidium* spp were detected in 15.5% and 3.3% of stool samples, respectively. Giardiasis and cryptosporidiosis were found in 3/3 and 2/3 day care centers, respectively, affecting mainly infants aged 13 to 24 months. A total of 8 *G duodenalis* isolates were confirmed as subassemblage BIV, all of them belonging to asymptomatic children. Attempts to genotype *Cryptosporidium* isolates failed. None of the variables considered could be associated with higher risk of infection with giardiasis or cryptosporidiosis. These results clearly indicate that asymptomatic infections with *G duodenalis* and *Cryptosporidium* spp are frequent in <3-year-old children in Central Spain.

## INTRODUCTION

The most common enteric protozoan pathogens affecting humans are *Giardia duodenalis* and *Cryptosporidium* species, which are major contributors to the burden of morbidity in the developed world.^1^ Direct person-to-person transmission of giardiasis and cryptosporidiosis is typically associated with poor fecal–oral sanitation and hygiene, although waterborne and foodborne transmission is also well documented worldwide.^2,3^ Additionally, international travelers returning from endemic areas and asymptomatic carriers may play an important role in the spreading of these infections.^4^

Although a significant percentage of cases of *G duodenalis* and *Cryptosporidium* infections may be asymptomatic, giardiasis and cryptosporidiosis typically result in diarrhea, with associated symptoms (eg, abdominal pain, nausea, vomiting, malabsorption, and weigh loss) ranging from acute to chronic.^5,6^ The severity of these diseases may be influenced by the parasite species/genotypes causing the infection and the age and immune status of the host. Therefore, in immunocompetent individuals, giardiasis is associated with intermittent symptomatology or even chronicity in many instances, whereas cryptosporidiosis is normally self-limiting and resolves spontaneously in 2 to 3 weeks. In immunocompromised subjects, cryptosporidiosis (but rarely giardiasis) may represent a life-threatening condition.^7^ This situation is further complicated by the fact that there is no vaccine or chemotherapeutical agent effective to prevent or treat cryptosporidiosis. Children attending day care settings and the elderly are among the most susceptible populations.

In Spain, *Giardia* and *Cryptosporidium* infections have been previously documented in a number of human, livestock, companion animal, and wild animal populations.^8,9^ However, reliable epidemiological information is restricted to certain geographical areas, whereas only incomplete or outdated information is currently available from most parts of the country. Molecular data regarding the species/genotypes circulating in Spain are even scarcer. Because of our limited knowledge on the frequency of giardiasis and cryptosporidiosis in populations of Spanish preschool children, the main goals of this study were to estimate the prevalence of *Giardia* and *Cryptosporidium* in children attending day care centers in Central Spain, molecularly characterize the parasites’ isolates obtained, and identify factors potentially associated with a higher risk of infection by these protozoan species.

## MATERIALS AND METHODS

### Area and Design of Study

The municipality of Majadahonda (Northwest of Madrid, Central Spain) has 70,198 inhabitants and extends >38.5 km^2^. Based on the 2012 census, there were 4664 (6.6%) children aged 0 to 4 years in its urban area. Migrant population accounted for 16.2% of the total population, with the highest proportion originating from South American and North-African countries. The municipality is endowed with 4 public day care centers located in districts of medium to high socioeconomic status, offering a total of 493 children’s places. Representatives of all 4 day care centers were personally contacted and, after holding informative meetings, asked for collaboration. Permission was obtained from 3 centers; the remaining one declined to participate in the study.

### Human Stool Samples and Questionnaires

A cross-sectional study was conducted in the spring of 2013 (April–June) among children (0–3 years old) attending 3 public day care centers in the municipality of Majadahonda. All children from each day care setting were invited to participate in the study. After informed consents were obtained from parents or legal guardians, recruited volunteers were provided with a prelabeled sampling kit including sterile polystyrene flasks for the recovery of stool samples, and instructions on how to take the sample safely. A standardized questionnaire covering demographic (age, sex, and day care center) data, clinical manifestations, contact with pet animals, visits to public parks or animal farms, and recent traveling abroad was also included. Collection of stool samples and epidemiological questionnaires were organized in collaboration with the day care centers at suitable times, transported to the laboratory at 4°C, and processed within 3 days after reception. No preservative solutions were added for long-term storage of stool samples.

For this study, cases were defined as children with stool samples positive for *G duodenalis* or *Cryptosporidium* by conventional microscopy and/or immunochromatography (ICT) and further confirmed by immunofluorescence microscopy. Symptoms considered compatible with giardiasis/cryptosporidiosis included diarrhea (defined as the occurrence of at least 3 watery stools within a 24-hour period), abdominal pain, constipation, nausea, and vomiting. This study has been approved by the research ethics committee of the Alfonso X El Sabio University.

### Conventional Microscopy for the Detection of *Giardia* and/or *Cryptosporidium*

In order to increase microscopy sensitivity, aliquots (1 mg) of all stool samples were processed and concentrated using routine coprological procedures including the merthiolate–iodine–formaldehyde solution and the modified Telemann method.^10^ Examination was conducted at ×200 magnification switching to ×400 magnification when structures morphologically compatible with *Giardia* cysts or *Cryptosporidium* oocysts were suspected.

### ICT Rapid Assay for the Detection of *Giardia* and/or *Cryptosporidium*

A strip of qualitative ICT commercial assay for the rapid simultaneous detection of *Cryptosporidium* and/or *Giardia* (Stick Crypto-Giardia; Operon, Zaragoza, Spain) was used in all stool samples. The tests were conducted at room temperature according to the recommended manufacturer’s instructions. Claimed diagnostic sensitivities and specificities of the test were 94% and 100% for *Cryptosporidium* and 100% and 95% for *Giardia*.

### DFATs for the Detection of *Giardia* and/or *Cryptosporidium*

Stool samples of children, which tested positive or probable for *Giardia* and/or *Cryptosporidium* by conventional microscopy and/or ICT, were further assayed by direct fluorescent antibody test (DFAT) for confirmation. Briefly, 5 μL of concentrated (as described above) stool samples were placed on welled slides. Smears were air-dried, methanol fixed, and stained with fluorescein-labeled mouse monoclonal antibodies (Crypto/Giardia Cel; Cellabs, Sydney, Australia). Samples were examined on a Zeiss fluorescence microscopy (Carl Zeiss, Oberkochen, Germany) equipped with a MC63 camera system (Carl Zeiss, Oberkochen, Germany) at ×400 magnification. Known positive and negative controls from clinical specimens submitted to our laboratory for diagnosis were routinely included in each sample batch.

### Total DNA Isolation

Total DNA was extracted from all DFAT-confirmed samples. A new, fresh aliquot (220 mg) of each stool sample was homogenized in stool lysis buffer and incubated at 95°C for 10 minutes. The DNA released from disrupted (oo)cysts was subsequently extracted using the QIAamp DNA Stool Kit (Qiagen, Hilden, Germany) according to the manufacturer’s instructions. Purified DNA samples (200 µL) were stored at −20°C until further analysis.

### Molecular Characterization of *G duodenalis* Assemblages

Extracted DNA from confirmed *G duodenalis*-positive samples were subsequently analyzed by multilocus sequence-based genotyping using 2 gene loci: glutamate dehydrogenase (*GDH*) and β-giardin (*BG*). The amplification of the *GDH* gene was performed by a seminested polymerase chain reaction (PCR) using the primer pairs GDHeF/GDHiF and GDHiF/GDHiF to yield a 432-bp fragment.^11^ The primary and secondary PCR reactions were carried out as follows: 1 step of 95°C for 3 minutes, followed by 35 cycles of 95°C for 30 seconds, 55°C for 30 seconds, and 72°C for 1 minute. A final extension of 72°C for 7 minutes and a 4°C hold was used.

The amplification of the *BG* gene was performed using a nested PCR using the primer pairs G7_F/G759_R and G99_F/G609_R to yield a 511-bp fragment.^12^ The primary PCR reaction was carried out with the following amplification condition: 1 step of 95°C for 7 minutes, followed by 35 cycles of 95°C for 30 seconds, 65°C for 30 seconds, and 72°C for 1 minute. A final extension of 72°C for 7 minutes and a 4°C hold was used. Cycling parameters for the secondary PCR reaction were the same as above except that the annealing temperature was 55°C. The list of oligonucleotide primers for the detection of *G duodenalis* at the *GDH* and *BG* loci are detailed in Supplemental Content 1 (http://links.lww.com/MD/A41).

PCR products were resolved on 2% D5 agarose gels (Conda, Madrid, Spain) stained with Pronasafe nucleic acid staining solution (Conda, Madrid, Spain). Amplicons of the expected size for *GDH* or *BG* were subsequently purified using the QIAquick Gel Extraction Kit (Qiagen, Hilden, Germany). In order to confirm *G duodenalis* assemblages, all purified amplicons were sequenced in both directions using the same internal primer sets as in their respective PCR assays.

### PCR-RFLP of the *GDH* and *BG*

Restriction digests were carried out on gel-purified PCR products in 20 µL reactions. Ten microliters of purified PCR product was added to 1× FastDigest buffer and 0.6 µL of FastDigest *Nla*IV and FastDigest *Rsa*I (for *GDH*), or 0.6 µL of FastDigest *Hin*dIII (for *BG*). All restriction enzymes were purchased from Thermo Fisher Scientific (Waltham, MA). Digestion was performed at 37°C for 4 hours or overnight. Restriction profiles were visualized on 2% agarose gels.

### Molecular Characterization of *Cryptosporidium* Species

Identification of *Cryptosporidium* species/genotypes was attempted by multilocus sequence-based genotyping using 2 gene loci: the small subunit *(SSU*) ribosomal RNA gene of *Cryptosporidium hominis* and the *Cryptosporidium* oocyst wall protein (*COWP*). Two nested PCR protocols were used to amplify the *SSU* and *COWP* as described elsewhere.^13,14^ The list of oligonucleotide primers for the detection of *Cryptosporidium* spp used are shown in Supplemental Content 1 (http://links.lww.com/MD/A41).

All *Giardia* and *Cryptosporidium* PCR reactions were carried out using BIOTAQ DNA polymerase (Bioline GmbH, Luckenwalde, Germany) on a 2720 thermal cycler (Applied Biosystems, Pleasanton, CA). Appropriate positive and negative controls were routinely included in each round of PCR.

### Sequence Analyses

Chromatograms and sequences were examined using the BioEdit sequence analysis program (http://www.mbio.ncsu.edu/BioEdit/page2.html). The BLAST tool (http://www.ncbi.nlm.nih.gov/blast/) was used to compare nucleotide sequences with sequences in the NCBI and GiardiaDB (http://giardiadb.org/giardiadb/) databases. Representative sequences for each *G duodenalis* assemblage at the *GDH* (GenBank accession numbers are L40509 (AI), L40510 (AII), AF069059 (BIII), L40508 (BIV), U60984 (C), U60986 (D), U47632 (E), and AF069057 (F)) and the *BG* (GenBank accession numbers are AY655702 (AI), AY072723 (AII), AY072724 (AIII), AY072727 (B), AY545646 (C), AY545647 (D), AY072729 (EI), AY545650 (EII), AY653159 (EIII), and AY647264 (F)) loci were used. Sequence alignments were conducted in MEGA 6 free software (http://www.megasoftware.net/).

### Data Analysis

To assess associations between possible risk factors measured in the questionnaire to children and infection with either *G duodenalis* or *Cryptosporidium* spp, data were analyzed with simple tails and prevalence odds ratios, and their 95% CI were calculated. Statistical power, defined as the probability of rejecting the null hypothesis while the alternative hypothesis is true, was calculated to determine the adequacy of the sample size used. Minimum power level was set at 0.80. Descriptive statistical analyses were conducted in OpenEpi, a free software (http://openepi.com/v37/Menu/OE_Menu.htm).

## RESULTS

**FIGURE 1:**
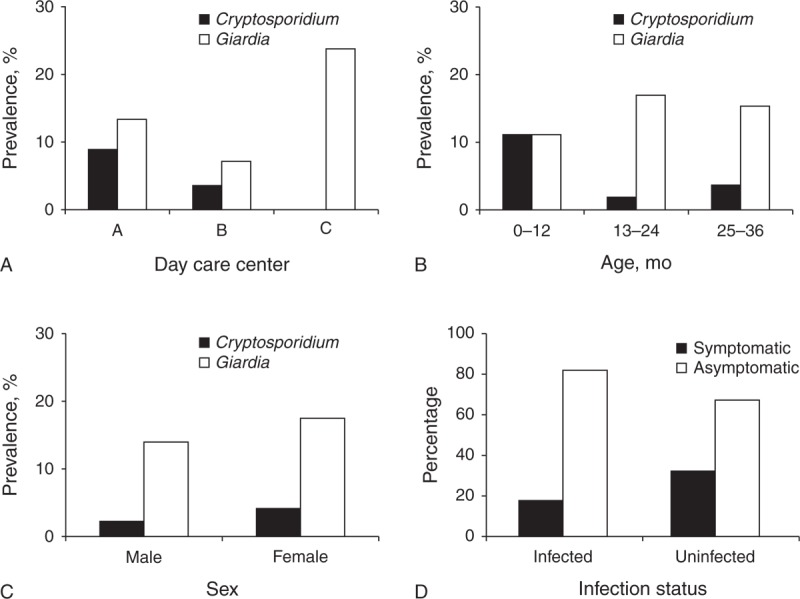
Presence of *G duodenalis* and *Cryptosporidium* spp infections in children by day care facility (A), group of age (B), sex (C), and presence/absence of symptoms compatible with cryptosporidiosis/giardiasis (D), Majadahonda, Central Spain.

A single stool sample was obtained from each of the 90 recruited children for a mean return rate of 20.9% (range, 19.2–25.2; SD, 3.4). Conventional microscopy, ICT, and DFAT showed matching positive results for *G duodenalis* in 14 (15.6%) samples, and for *Cryptosporidium* spp in 3 (3.3%) samples, respectively. Epidemiological questionnaires were satisfactorily completed and returned by 88 participants (response rate, 97.8%) and considered in the analyses (Table 1). The male:female ratio was 0.91. The age range was from 10 to 36 months (mean, 24.6; SD, 7.8), with 13 to 24 month children accounting for 60.2% of the total. *G duodenalis* and *Cryptosporidium* spp infections were detected in 3/3 and 2/3 day care centers (Figure 1A). Both *G duodenalis* and *Cryptosporidium* spp affected children of all age ranges, with the former presenting higher prevalence in infants of 13 to 36 months of age (Figure 1B). Children of both sexes were similarly affected by cryptosporidiosis or giardiasis (Figure 1C). Interestingly, only 3/17 (17.6%) cases infected with *G duodenalis* or *Cryptosporidium* spp presented symptoms clinically compatible with infections caused by these pathogens (Figure 1D).

**TABLE 1:**
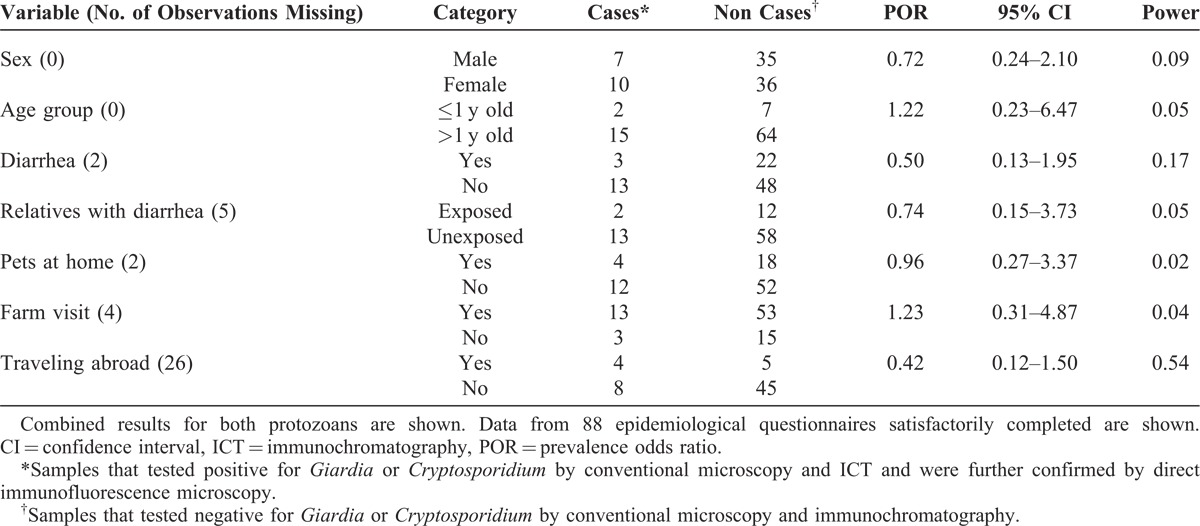
Analysis of the Variables Included in the Epidemiological Questionnaire Potentially Involved in the Transmission of *Giardia* and/or *Cryptosporidium*

All children had been visiting parks and public playgrounds regularly before the survey. Twenty two children including 3 *G duodenalis* cases and 1 *Cryptosporidium* case reported having pets (dogs, cats, turtles, canaries, and parakeets) at home. Only 9 children (5.7%) traveled to a foreign country during the past 6 months, mainly in Europe (France, Italy, Portugal, and Romania) and South America (Brazil). None of the variables considered in this study (day care center, sex, age, contact with relatives experiencing diarrheal episodes, contact with pets, farm visits, and traveling abroad) could be associated with higher prevalence odds of infection with cryptosporidiosis or giardiasis (Table 1). The low statistical power of the analyses suggests our data may not detect such associations due to small sample size and, in the case of *Cryptosporidium*, small number of positive results.

Out of the 14 samples positive for *G duodenalis*, 8 isolates were successfully amplified at the *GDH* locus. Digestion of purified PCR products with *Nla*IV resulted in identical profiles, suggestive of subassemblage BIII or BIV^11^ (Supplemental Content 3, panel A, http://links.lww.com/MD/A43). Failure to cleave the original PCR products with *Rsa*I (Supplemental Content 3, panel B, http://links.lww.com/MD/A43) confirmed all 8 isolates as subassemblage BIV.^11^ A 366-pb fragment, equivalent to positions 103 and 468 of the subassemblage BIV reference sequence (GenBank accession number, L40508), were cleanly read from the raw sequence data for each sequence. Alignment analyses revealed 100% similarity among 6 of our isolates (isolates 143, 150, 156, 157, 160, and 167) and the reference sequence L40508. The remaining 2 sequences (isolates 145 and 158) presented 4-point mutations at positions 183 (T to C), 387 (T to C), 396 (C to T), and 423 (C to T).

The *BG* gene was successfully amplified in 7/8 isolates already amplified at the *GDH* locus. Digestion of purified PCR products with *Hae*III resulted in identical profiles (Supplemental Content 3, panel C, http://links.lww.com/MD/A43) compatible with assemblage B.^11^ Alignment analyses of the *BG* sequences for these samples demonstrated that all 7 isolates (isolates 145, 150, 156, 157, 158, 160, and 167) had identical sequences, but revealed 6-point mutations at position 159 (G to A), 165 (C to T), 309 (C to T), 324 (C to T), 393 (C to T), and 471 (T to C) when compared with a 437-pb fragment equivalent to positions 137 and 573 of the corresponding assemblage B reference sequence (GenBank accession number, AY072727).

Representative sequences from each different isolate found in this study were submitted to GenBank, including isolate 158 (accession number, KJ645940) and isolate 145 (accession number, KJ645941) for *GDH*, and isolate 150 (accession number, KJ645942) for *BG*, respectively.

Unfortunately, attempts to amplify the *SSU* and *COWP* genes in all 3 samples positive for *Cryptosporidium* spp failed repeatedly.

## DISCUSSION

Infants and toddlers are particularly susceptible to oral–fecal transmitted infectious diseases, presumably because of their immature and inexperienced immune systems, high hand-to-mouth activity, and undeveloped hygienic habits. Childcare facilities might provide adequate environments for the fast spread of enteric infections with children confined within limited spaces, particularly if appropriate sanitation and hygiene standards are not fulfilled.^15^ It is, therefore, not surprising that day care center attendants have increased risk of acquiring childhood diseases.^16,17^ Among them, both symptomatic and asymptomatic infections by *G duodenalis* and *Cryptosporidium* spp have been frequently reported in day care centers in developed countries including Germany, The Netherlands, and the United Kingdom.^18–20^

In Spain, previous epidemiological studies in pediatric populations have demonstrated the presence of *G duodenalis* and *Cryptosporidium* spp infections in 3% to 25% and 1% to 10% of the children analyzed, respectively.^8,9,21,22^ Therefore, the frequency of *G duodenalis* (15.6%) and *Cryptosporidium* spp (3.3%) found in the present study falls well within the prevalence ranges documented in previous national surveys. However, these data should be interpreted with caution due to the comparatively small sample size of this study. Additionally, it is important to take into consideration that these figures may underestimate the actual prevalence of these pathogens because of 2 potential drawbacks. First, the screening tests used in our study (conventional microscopy and ICT) may fail to detect infections at very low intensities. Second, because *G duodenalis* and *Cryptosporidium* spp shed (oo)cysts intermittently and only a single stool specimen was examined per child, it is likely that a number of infections had been missed.

Importantly, most (82.4%) of the children with giardiasis or cryptosporidiosis did not show any clinical manifestation of illness. This finding is in agreement with the data obtained in similar studies in other European countries.^19,20^ Because asymptomatic carriers serve as a community reservoir of disease, they are potential contributors to the spreading of the infection to healthy subjects, including adults. Indeed, child-to-adult infection transmission of *G duodenalis* seems plausible as housewives and nursing mothers changing nappies have been demonstrated to have a 4-fold increased risk of giardiasis.^17^

Although not completely elucidated, it seems clear now that the pathology and virulence of *G duodenalis* and *Cryptosporidium* spp infections are the consequence of a multifactorial process involving both host (age, immune system status, and nutritional status) and parasite (strain genotype, infectious dose, and coinfections) features.^6,7,23^ In recent years, a number of studies have attempted to correlate the presence of clinical manifestations (mainly diarrhea) with the genotype of the parasite causing the infection. In the case of *G duodenalis*, an early study carried out in The Netherlands found that *G duodenalis* assemblage A isolates were more prevalent in asymptomatic infected individuals, whereas assemblage B isolates were more frequently found in infected subjects with persistent diarrhea.^24^ Opposite results have been consistently reported in surveys carried out in Australia, Bangladesh, Portugal, Spain, and Turkey.^25–29^ Inconclusive data or no correlation between genotypes and symptoms were obtained in other studies performed in Brazil, India, and Iran.^30–32^ Molecular information from Spanish pediatric populations have shown that, in patients <5 years old, symptomatic giardiasis was present in 81.2% of assemblage AII infections but only in 34.6% of assemblage B cases.^28^ In another study, assemblage B was the genotype chiefly found (57.1%) in apparently healthy children of 1 to 12 years.^33^ In the present survey, all 8 isolates of *G duodenalis* from 8 different asymptomatic children were conclusively assigned to subassemblage BIV. The fact that subassemblage BIV was found circulating in the 3 day care centers under study indicates that this may be the most prevalent *G duodenalis* genotype in children in this geographical area. Although limited, our results also support the hypothesis that assemblage B isolates are predominantly found in infected subjects without clinical manifestations.

This is the first study describing *G duodenalis* assemblage B subtypes in Spanish human isolates. Based on sequence analyses of the *GDH* gene, a genetic variant (accession number, KJ645941) was identified in 2/8 isolates characterized as subassemblage BIV. This variant had 100% similarity with partial sequences reported in human isolates from Holland (accession number, AY826197), Norway (accession number, DQ923581), and Thailand (accession number, HM747963). Similarly, based on sequence analyses of the *BG* gene, all 7 isolates characterized were assigned to the same *G duodenalis* assemblage B variant (accession number, KJ645942). This variant showed 100% similarity with partial sequences reported in human isolates from Belgium (accession number, EU881698), Sweden (accession number, HM165208), Egypt (accession number, HM171691), Australia (accession number, HQ179581), and New Zealand (accession number, EU274397). Taken together, these data seem to suggest that the *G duodenalis* assemblage B genotypes/subtypes described in the present study have very likely a global distribution.

Regarding *Cryptosporidium* infections, among the 173 *Cryptosporidium* isolates characterized in Spanish children to date, *C hominis* (62.4%) was the *Cryptosporidium* species more frequently identified, followed by *C parvum* (35.3%), **C* meleagridis * (1.7%), and *C felis* (0.6%).^34,35^ Unfortunately, in this study, amplification of *Cryptosporidium*-specific DNA from infected children failed and no genotyping could be carried out. The fact that very low numbers of oocysts were detected by DFAT in the 3 *Cryptosporidium*-positive samples suggests that very likely the amount of purified DNA was beyond the detection limit of the PCR methods used. Therefore, the *Cryptosporidium* species currently circulating in this pediatric population remains unknown.

In summary, our epidemiological and molecular data reveal that both *G duodenalis* and *Cryptosporidium* spp infections are frequent in children attending day care facilities in Central Spain. The finding that 3 quarter of the children infected with these pathogens did not have clinical manifestations raises important public health concerns, as asymptomatic carriers may inadvertently spread these diseases to other community members. This fact highlights the convenience of carrying out universal screening programs aiming to detect asymptomatic giardiasis and cryptosporidiosis in order to minimize the possibility of child-to-child or child-to-adult transmission. Finally, well-designed case–control studies are needed to undoubtedly demonstrate the potential correlation between a specific *G duodenalis* assemblage and the development of associated clinical features in infected individuals.

## ACKNOWLEDGMENT

The authors thank Dr Israel Cruz (National Centre for Microbiology, Majadahonda, Spain) for assisting with sequence analyses.
